# Bidirectional Interplay Between Traumatic Brain Injury and Cardiovascular Dysfunction in Athletes

**DOI:** 10.3390/jcm14217712

**Published:** 2025-10-30

**Authors:** Fazle Kibria, Olga A. Bragina, Alex O. Trofimov, Denis Bragin

**Affiliations:** 1 Lovelace Biomedical Research Institute, Albuquerque, NM 87108, USA; obragina@lovelacebiomedical.org (O.A.B.); dbragin@lovelacebiomedical.org (D.B.); 2 Department of Neurological Diseases, Privolzhsky Research Medical University, 603005 Nizhny Novgorod, Russia; xtro7@mail.ru; 3 Department of Neurology, The University of New Mexico School of Medicine, Albuquerque, NM 87106, USA; 4 Department of Physiology, New York Medical College, Valhalla, NY 10595, USA

**Keywords:** heart failure, brain injury, sports, neurocardiology in sports, sports injury, CVD

## Abstract

Sports-associated traumatic brain injury is emerging as an under-recognized driver of acute and chronic cardiovascular diseases. Larger population-based studies show that individuals with moderate-to-severe traumatic brain injury experience up to a two-fold excess risk of incident hypertension, coronary artery disease, myocardial infarction, and stroke that persists for at least a decade. Among former professional American-style football players, a higher lifetime concussion burden is uniquely related to a more atherogenic cardiometabolic profile and greater long-term stroke risk. Mechanistically, an acute “sympathetic storm” triggered by cerebral injury provokes catecholamine surges, endothelial dysfunction, and myocardial stunning, manifesting as neurogenic stunned myocardium or Takotsubo-like cardiomyopathy and malignant arrhythmias. Sub-acute to chronic phases are characterized by persistent autonomic imbalance, reflected by reduced heart-rate variability and impaired baroreflex sensitivity weeks to months after concussion, coupled with neuroinflammation, hypothalamic–pituitary–adrenal axis dysregulation, and lifestyle changes that accelerate atherosclerosis. The interplay of these pathways accounts for the elevated burden of cardiovascular disease observed long after neurological function has been restored. Despite robust evidence linking TBI to adverse cardiac outcomes, contemporary sports–cardiology risk stratification prioritizes hemodynamic load, genetics, and performance-enhancing substances, largely overlooking brain injury history. This review integrates epidemiological, clinical, and mechanistic data to (i) delineate acute neurocardiac complications secondary of sports-related traumatic brain injury, (ii) synthesize evidence for chronic cardiovascular risk, (iii) highlight emerging autonomic and inflammatory biomarkers, and (iv) propose surveillance and therapeutic strategies, ranging from heart-rate-variability-guided return-to-play decisions to aggressive cardiometabolic risk modification aiming to mitigate long-term morbidity in this athletic population. By framing sports-related traumatic brain injury as a modifiable cardiovascular risk factor, we aim to foster interdisciplinary collaboration among neurologists, cardiologists, and sports medicine practitioners, ultimately improving both neurological and cardiovascular outcomes across the athlete’s lifespan.

## 1. Introduction

Sport is integral to youth development and confers broad health dividends when practiced safely. Engagement in regular athletic activity is associated with significant improvements across multiple psychological domains, including enhanced self-esteem, cognitive function, social competence, and overall life satisfaction, as well as reduced symptoms of depression, anxiety, and stress [1,2,3]. At the cerebral level, exercise stimulates synaptogenesis, angiogenesis, and neuroplasticity through enhanced cortical perfusion and neurotrophic signaling [4,5,6]. Cardiorespiratory training promotes endothelial nitric oxide synthase (eNOS)-dependent mitochondrial biogenesis and augments myocardial glucose uptake, thereby reducing the incidence and recurrence of cardiovascular disease (CVD) [7,8,9].

However, these psychological benefits can be substantially undermined by the occurrence of sport-related injuries. Children ≤14 years sustain >3.5 million sports- or recreation-related injuries annually in the United States alone [10]. Cardiac complications—including fatal ventricular arrhythmias and exercise-induced myocardial ischemia underlie much of the reported sudden cardiac death in athletes [11,12,13,14,15], accounting for ≈2000 deaths each year in Americans <25 years [16]. Head trauma is highly prevalent: ≈21% of all traumatic brain injuries (TBIs) arise from athletic or recreational activities [10], and an estimated 3.8 million sports-related concussions occur annually, half of which go unreported [17].

Growing evidence links TBI to both acute and chronic CVD. Proposed mediators include autonomic dysregulation [18], systemic inflammation [19,20], catecholamine surges [21], and altered chemo/baroreceptor function in response to injury-related ventilatory demands [22,23,24]. The autonomic nervous system, which orchestrates heart rate, respiration, digestion, and thermoregulation [25,26], can shift toward sympathetic activation after TBI, a pattern associated with tachyarrhythmias, blood-pressure lability, and heightened long-term cardiovascular risk [27,28]. A brain injury triggers a localized inflammation in the brain and a systemic inflammation that can affect the entire body [29]. Post-TBI systemic inflammation has been shown to be associated with a peripheral shift in immune capacity [30] and the associated risk of cardiac impairment [31,32]. Excess catecholamines aggravate cardiomyocyte cytotoxicity, elevate myocardial oxygen demand, and provoke coronary vasospasm [33,34,35]. Disrupted chemoreflex and baroreflex control further destabilize cerebral and cardiac hemodynamics [36,37].

Extensive experimental evidence and clinical findings revealed a hidden relationship between the heart and the brain [38]. Acute brain injuries, such as ischemic stroke [39], TBI [40], or aneurysmal subarachnoid hemorrhage [41], can result in arrhythmias, neurogenic stress cardiomyopathy, and heart failure (HF) even in previously healthy individuals [42,43,44,45]. Conversely, primary cardiac dysfunction may exacerbate cerebral ischemia, hypoperfusion, and reperfusion injury, compounding neurological damage [46,47,48,49,50].

Despite these observations, the cardiovascular sequelae of sport-related brain injury remain understudied. This review, therefore, addresses two key questions: (i) How does athletic TBI influence cardiac structure and function? and (ii) To what extent do injury-associated cardiac abnormalities impair neurological recovery and performance in athletes? By synthesizing current clinical, experimental, and mechanistic evidence, we aim to clarify the bidirectional brain–heart impairment that follows sport-related injury and to identify priorities for future interdisciplinary research.

## 2. Methods

We conducted a literature review to map the bidirectional relationship between sport-related brain injury and cardiovascular dysfunction. PubMed (MEDLINE) and Google Scholar were searched with a Boolean string combining (“brain injury” OR “concussion” OR “traumatic brain injury”) AND (“sport*” OR “athlete*”) AND (“cardiac” OR “cardiovascular” OR “heart” OR “arrhythmia*” OR “myocardial” OR “stroke” OR “baroreflex” OR “auto-nomic”), iteratively augmented with terms such as “neurogenic stress cardiomyopathy,” “catecholamine surge,” and “systemic inflammation”. Reference lists of eligible articles and pertinent reviews were hand-searched to ensure completeness. We included English-language studies—randomized trials, cohort, case–control, cross-sectional, case series (≥3 subjects), and relevant animal models—that reported acute (≤30 days) or chronic (>30 days) cardiovascular outcomes after a documented concussion, mild-to-severe TBI, or repetitive head impacts sustained during sport or recreation. Other injuries without head involvement, non-sport trauma, non-English papers, editorials, conference abstracts, and single-patient case reports were excluded. Two reviewers independently screened titles/abstracts and full texts and extracted data into a pre-piloted spreadsheet, and disagreements were resolved by consensus or a third reviewer. Owing to heterogeneity in populations, exposure definitions, and outcome metrics, quantitative pooling was inappropriate; instead, findings were synthesized narratively across four domains—epidemiology, acute neuro-cardiac complications, chronic cardiovascular sequelae, and reverse brain-heart interactions—with particular attention to consistent effect directions, sport-related concussion and cardiovascular dysfunction, dose–response patterns, cardiac contribution to brain dysfunction, and unresolved knowledge gaps.

## 3. Cardiovascular Benefits and Risks of Sports Participation

Regular participation in structured physical activity remains one of the most potent non-pharmacological strategies for promoting lifelong health. In the central nervous system, exercise enhances synaptogenesis, cerebrovascular perfusion, neurotrophic signaling, and neuroplasticity, thereby sharpening cognition and executive function [51]. Systemically, it supports musculoskeletal integrity and optimizes cardiovascular, endocrine, and immune networks [52,53,54], translating into lower lifetime incidence of neoplasms, cardiometabolic disease, and frailty [55,56,57]. Conversely, lower physical activity is now recognized as a leading modifiable driver of global mortality and CVD burden [58]. In contrast, individuals who meet guideline-recommended activity thresholds demonstrate a consistently lower CVD risk profile [59]. Moreover, the World Health Organization projects a US$300 billion increase in healthcare expenditure between 2020 and 2030 if current inactivity trends persist [60].

**Figure 1:**
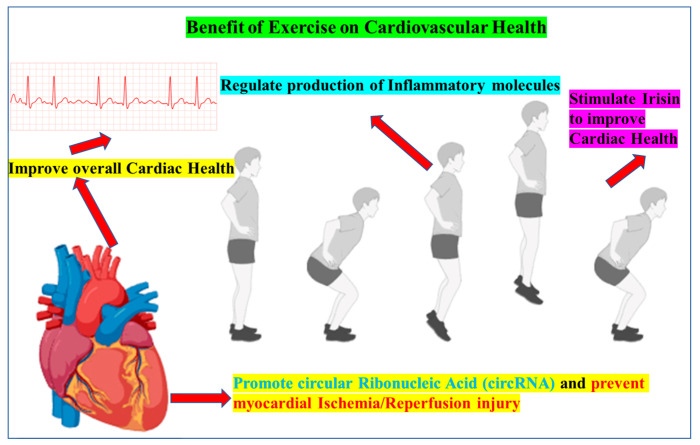
Exercise-Induced Cardioprotective Pathways. Regular physical activity improves myocardial contractility and coronary perfusion, dampens systemic and myocardial inflammation, and limits maladaptive remodeling. Two molecular mediators highlighted here are (i) exercise-responsive circular RNAs, which modulate pro-inflammatory and cell-death signaling during ischemia–reperfusion (I/R), and (ii) the myokine irisin, whose release from working skeletal muscle boosts eNOS activity and mitochondrial function. Together, these mechanisms confer resistance to myocardial I/R injury and support long-term cardiovascular health.

Physical exercise is known to confer primary and secondary cardioprotection via multiple converging mechanisms. Habitual training lowers blood pressure, fasting glucose, insulin, and body mass index, while improving cardiorespiratory fitness [9,61]. Aerobic conditioning augments eNOS activity, stimulates mitochondrial biogenesis, and bolsters cardiac glucose uptake—effects believed to counteract age-related CVD progression [62]. Working skeletal muscle releases myokines, including the recently described fibronectin type III domain-containing protein 5-cleavage product irisin, which exerts salutary paracrine effects on myocardial metabolism and contractility [63,64]. Resistance training, when properly periodized, is equally cardioprotective in both healthy adults and patients with established CVD [65], and even a 12-month program can meaningfully attenuate CVD risk factors in long-term childhood cancer survivors [66]. Pre-clinical and translational data further show that exercise upregulates cardioprotective circular ribonucleic acid (RNAs) [67], preserves vascular-endothelial telomere length, and limits senescent-cell-driven inflammation [68]. In women, lifelong activity appears to mitigate the adverse vascular remodeling and protein shifts that accompany estrogen loss at menopause [69]. The inset Figure 1 represents the overall cardiac benefits of exercise.

These clinical benefits are underpinned by a cascade of molecular adaptations: (i) reduction in traditional cardiometabolic risk factors [70,71]; (ii) attenuation of myocardial I/R injury via optimized coronary perfusion, reduced endoplasmic-reticulum stress, cyclo-oxygenase-2 induction, heat-shock-protein activation and enhanced sarcolemma/mitochondrial adenosine triphosphate (ATP)-sensitive potassium (K^+^)-channel activity [72,73,74]; (iii) limitation of pathological cardiac hypertrophy through phosphorylated-histone-H3 signaling [75,76]; (iv) eNOS-mediated nitric-oxide release that cushions the heart against acute ischemic insults [77,78]; (v) improved ATP production via enhanced calcium (Ca^2+^)-dependent ATPase and reduced form of nicotinamide adenine dinucleotide oxidation [79,80,81]; (vi) mobilization of extracellular vesicles that convey cardioprotective RNA and protein cargos [82,83]; and (vii) stimulation of endogenous cardiomyocyte renewal, thereby reinforcing myocardial regenerative capacity [84].

Yet sports participation is not risk-free. Sudden cardiac arrest (SCA) remains the leading cause of death in young athletes. Typically precipitated by ventricular arrhythmia or ischemia that compromises cerebral and systemic perfusion [11,12,85,86,87,88,89,90]. Hypertrophic cardiomyopathy alone accounts for ≈36% of these events [91]. Long-QT syndrome, arrhythmogenic right ventricular cardiomyopathy, and anomalous coronary-artery physiology constitute other major substrates [92]. Inherited cardiomyopathies and ion-channelopathies together underpin most SCA cases in this demographic [91,93], although their precise molecular triggers are incompletely understood. Intense or prolonged bouts can also elevate cardiac biomarkers such as cardiac troponin-T, brain natriuretic peptide, and D-dimer [94,95]. The prognostic significance of these transient surges remains uncertain. Indeed, some epidemiological and mechanistic studies suggest a J- or U-shaped curve in which very high cumulative exercise loads may paradoxically heighten atrial fibrillation risk or accelerate coronary artery calcification [96]. Defining the boundary between adaptive and maladaptive cardiac responses to sport, therefore, remains an important research priority. Particularly, it intersects with the emerging evidence that sport-related brain injury can further modulate cardiovascular vulnerability.

## 4. The Neurocardiac Consequences of Sports-Related Brain Injury

It is reported that approximately 10% of all brain injuries in the United States are attributable to sports and recreational activities, with children and adolescents accounting for 21% of these cases [97]. Contact sports such as boxing, rugby, American football, wrestling, and basketball account for most of these events and expose athletes to both single and repetitive TBI or concussion. Growing epidemiological evidence links such head impacts to a heightened lifetime risk of chronic cardiovascular disease, an association also documented in military personnel and retired professional football players who share high cumulative concussion burdens [23].

### 4.1. Experimental and Clinical Evidence

**Figure 2:**
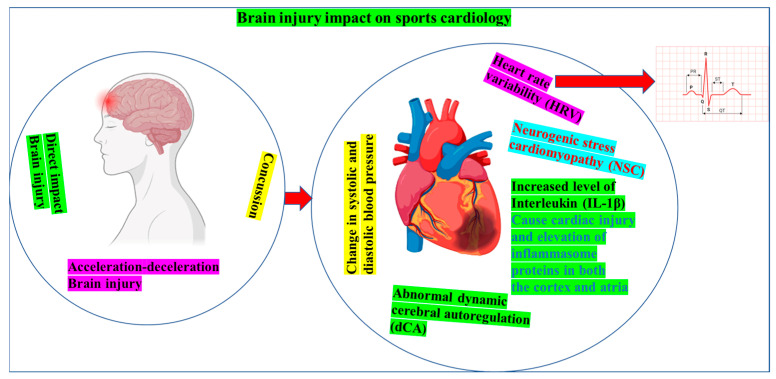
Neuro-cardiac sequelae of sport-related brain injury. Traumatic or repetitive concussive impacts initiate sympathetic surges and inflammasome-driven inflammation, elevating interleukin-1β and related cytokines. The resulting autonomic imbalance decreases HRV, produces labile systolic/diastolic blood pressure, and disrupts dynamic cerebral autoregulation. In some athletes, these cascades culminate in NSC and heighten the risk of acute arrhythmias and chronic myocardial injury.

Preclinical studies on juvenile mice subjected to mild traumatic brain injury demonstrated an immediate reduction in cerebrovascular oxygen saturation and elevated hemoglobin concentrations in the affected left cortical region [98], which indirectly affects cardiac function. These cerebral changes are paralleled by impaired diastolic relaxation, attenuated systolic strain, depressed myocardial performance, and an unfavorable shift in cardiac-biomarker profiles [98]. The initial cerebrovascular hypoxia and chronic cardiac dysfunction are associated with long-term behavioral changes [98]. In human cohorts, 25–35% of patients with moderate-to-severe TBI develop QT-interval prolongation, supraventricular arrhythmias, regional wall motion abnormalities, troponin elevation, or transient myocardial stunning [99]. Mechanistically, TBI provokes a systemic inflammatory response [30] and a catecholamine surge [100] that disrupts autonomic balance, activates endothelial and immune pathways, and precipitates cardiomyocyte injury [101,102,103]. Up-regulation of inflammasome proteins, including absent in melanoma 2, apoptosis-associated speck-like protein, and caspases-1/8/11, in both the cortex and the atria, with parallel elevations in interleukin-1β, has been demonstrated in murine TBI models and is accompanied by the release of pro-inflammatory extracellular vesicles that alter cardiac biology [19,104,105]. In clinical settings, psychiatric comorbidity, chronic pain, and cardiovascular impairment often co-occur after mild TBI, with older patients displaying greater cardiac vulnerability [106,107]. Children with severe TBI exhibit elevated QT and ST shortening in heart-rate variability (HRV) and impaired autonomic control [108]. The HRV deficits also characterize adults with cognitive impairment, dementia, and prior brain injury [109]. Neurogenic stress cardiomyopathy (NSC) is a well-recognized acute complication triggered by cerebral insults [110], and elevated cardiac troponin plus abnormal echocardiography (left-ventricular ejection fraction < 50% or regional wall-motion defects) are frequent after TBI [111]. Collectively, these data implicate autonomic disequilibrium, systemic inflammation, and catecholamine toxicity as primary drivers of post-TBI cardiac dysfunction [112,113]. Cardiorespiratory deconditioning and exercise intolerance that follow TBI further amplify long-term CVD risk [114,115]. The pathophysiological pathways linking brain injury to cardiac impairment are summarized in Figure 2. Despite clear mechanistic links, no single biomarker has yet unified brain–heart injury into a clinically actionable metric, and the relative contributions of mild, moderate, and severe TBI in athletes remain under investigated [116]. Alternatively, emerging evidence indicates that acute non-traumatic brain injury can induce discernible electrocardiographic alterations, further intensifying research interest in neuro-cardiac interactions.

### 4.2. Sport-Related Concussion and Cardiovascular Dysfunction

Mild traumatic brain injury accounts for ≈95% of all TBIs and is at least 18-fold more common than moderate-to-severe forms [117]. Concussion, defined as a transient neurological disturbance induced by biomechanical forces to the head or body, disrupts neurotransmission, alters cerebral metabolism, and impairs axonal homeostasis [118,119]. Incidence, symptom burden, and recovery trajectories vary by sport, age, and access to specialist care [120,121,122]. Young athletes are particularly vulnerable because brain maturation and motor-system development are ongoing [123,124]. The sports-related consequences of systematic changes are discussed in the upcoming sections.

### 4.3. Acute Autonomic and Hemodynamic Changes

Immediately after SCA, fluctuations in heart rate and changes in systolic/diastolic blood pressure are common [125]. Cardiac-cycle analysis reveals shortened systolic duration and prolonged atrial systole, suggesting altered contractility [126]. Autonomic imbalance manifests as reduced HRV and impaired dynamic cerebral autoregulation [127]. Sympathetic overdrive raises ventilatory demand, stiffens arteries, and can elevate blood pressure [37,128]. In summary, SCA induces several changes in acute autonomic responses, including a sympathetic surge, parasympathetic withdrawal, arrhythmia susceptibility, and altered hemodynamics, which contribute to myocardial dysfunction, impaired vasodilation, and impaired microcirculation, ultimately worsening patient outcomes [129,130,131].

### 4.4. Chronic Sequelae

Retired professional football players with repetitive concussions show higher rates of atherogenic profiles, coronary disease, and stroke decades later [132,133]. Repeated sports-related concussion (SRC) promotes neuro-autonomic cardiovascular abnormality, with lingering HRV, depression, exercise intolerance, and baro-/chemoreflex dysregulation [134,135]. Biological sex modulates HRV patterns and recovery trajectories, underscoring concussion heterogeneity [136,137]. Individuals sustaining multiple concussions within five years may present with persistent autonomic dysfunction, vestibular and oculomotor deficits, cognitive decline, and chronic headache—features often accompanied by cardiac and vascular abnormalities [138]. The series of interrelated incidents after a concussion degraded the systematic and functional control of neurocardiac reversibility, inviting long-term health issues as a chronic sequel.

### 4.5. Putative Mechanisms

Post-SRC cardiovascular impairment arises through: (i) autonomic shift and HRV depression [37]; (ii) arterial stiffening and blood-pressure lability [128]; (iii) hypoxia-, hypercapnia- and pH-driven chemo-/baroreceptor activation [37,139]; (iv) sympathetic stress that shortens systolic timing and depresses myocyte performance [140]; (v) altered afferent–efferent cardiac-nerve signaling [37]; and (vi) neurometabolic crisis with mitochondrial dysfunction that increases cardiac-output demand [37]. Psychological sequelae—anxiety, fear, and depression—add a further cardiometabolic load and warrant routine screening during follow-up [37,141].

Emerging literature also illustrates the opposite limb of the brain–heart axis: cardiac pathology can exacerbate cerebral ischemia, neurodegeneration, and cognitive decline [142,143,144,145]. A comprehensive evaluation of athletes with concussion should therefore include cardiovascular screening, autonomic testing, and longitudinal monitoring to detect delayed neuro-cardiac complications, as represented in Table 1.

## 5. Cardiac Contribution to Brain Dysfunction

The heart–brain axis is bidirectional—the same autonomic, immune, and neurohormonal pathways that transmit injury signals from the brain to the heart also allow cardiac pathology to disrupt cerebral homeostasis [146,147]. Clinical and experimental work now shows that common cardiovascular disorders in athletes—including HF, myocardial infarction (MI), arrhythmias, coronary artery disease, and the cardiometabolic cluster of metabolic syndrome—can accelerate cognitive decline and increase dementia risk through four inter-related mechanisms: (i) persistent cerebral hypoperfusion and hypoxia, (ii) cardio-embolic or thrombotic brain injury, (iii) systemic and neuro-inflammation, and (iv) chronic neuro-hormonal activation [146,148,149]. The most common cardiac impairment and its association with brain abnormality are described in the following section.

### 5.1. HF

The progression of dilated cardiomyopathy or chronic pressure/volume overload to HF in athletes is linked to significant comorbidities, including cognitive impairments (learning and working-memory deficits), psychological disorders (anxiety and depression), and a decline in sport-specific motor skills [150,151]. An average of 40% HF patients suffer from improper memory, difficulties in concentration, attention deficits, and cognitive impairment [152]. HF reduces cerebral blood flow, particularly to the hippocampus, amygdala, and prefrontal cortex, provoking hypoxic injury, microglial activation, and grey-matter atrophy that underlie cognitive and emotional disturbances [153,154,155,156]. Rodent models confirm microglial polarization toward a pro-inflammatory phenotype and impaired synaptic plasticity in the dorsal hippocampus proportional to HF severity [157,158]. HF leads to reduced heart pump efficiency and decreased oxygen supply to the brain, as demonstrated in a patient study, which affects frontal, parietal, and medial cortex, gray matter density, and higher levels of N-terminal prohormone of brain natriuretic peptide—a biomarker used for screening, diagnosis, and prognosis of HF [159]. Studies suggested that neurocardiac pathophysiological pathways may be significant in understanding neuropsychiatric symptoms in athletes with heart disease. However, further research evidence on sports-related HF has yet to be studied to explore the area.

### 5.2. MI

Decreased blood flow in the coronary artery due to obstruction/occlusion of the vessels leads to MI. Post-MI patients experience steeper declines in global cognition, memory, and executive function than age-matched controls [160,161,162,163]. Proposed drivers include transient cerebral hypoperfusion during the infarct, systemic inflammatory spill-over, and accelerated atherosclerosis. It is well established that MI can induce long-term neuroinflammation that may persist beyond the initial peripheral inflammation. As evidence, the rat’s hypothalamus demonstrates significant neuroinflammation approximately 6–8 weeks after MI [164]. Neuroinflammation is a key factor in multiple brain abnormalities, including the progression of Alzheimer’s dementia, behavioral problems, anxiety, cognitive deficit, and depression [165]. An MI in a sports person can impact lifelong disability in performance and activity.

### 5.3. Arrhythmias

Cardiac arrhythmia, a rhythmic disorder caused by irregular electrical activity originating from the heart and disrupting the physiological synchronization of cardiac contractions, is a medical condition where the heartbeat shows irregular rhythms, ranging from slow (>60 beats/min) to fast (>100 beats/min—tachyarrhythmias), and can be present at any age [11,12]. Atrial fibrillation is the most common type of cardiac arrhythmia, affecting 33.5 million people worldwide, and is an independent risk factor for acute ischemic stroke [166]. Several studies reported that arrhythmia can compromise the performance and longevity of athletes’ skills and has long-term adverse effects if not treated promptly [167,168,169]. Atrial or ventricular tachyarrhythmias commonly observed in endurance athletes can precipitate dementia via cerebral hypoperfusion, micro-emboli, inflammation, and overt or silent stroke [166,170,171]. Each episode of atrial fibrillation compounds the risk. Athletes with atrial fibrillation-related stroke show the most tremendous cognitive loss. A study demonstrated that athletic activities are associated with a proarrhythmic heart [163], suggesting that regular examination of heart parameters in athletes is crucial to maintain their well-being and optimal performance.

### 5.4. Metabolic Syndrome and Coronary Disease

Metabolic syndrome causes an imbalance between energy intake and energy expenditure, leading to an increased risk of cardiovascular and cerebrovascular diseases. Visceral adiposity, dyslipidemia, and insulin resistance impair cerebrovascular reactivity, damage white matter, and promote anxiety and depression [165,166,167,168,169]. Increased free radical production, neuroinflammation, reduced levels of neurotrophic factors, and reduced insulin transport into the brain have been reported in patients with metabolic syndrome [170,171]. Evidence indicates that biochemical features of metabolic syndrome, such as systemic impairment of antioxidant and higher lipid peroxidation, increase the risk of stroke [172,173]. Coronary artery disease (CAD) further weakens functional connectivity between bilateral thalami and multiple cortical hubs, a pattern that parallels small-vessel disease and cognitive slowing [174,175]. A longitudinal study of the historical suffering of CAD patients revealed changes in white matter hyperintensities, silent brain infarcts, and cortical gray matter, leading to cognitive declines in global cognition, verbal memory, and executive function [176]. While exercise is beneficial for cognitive health, athletes with metabolic syndrome and coronary disease might not experience the same level of protection. Studies indicated that individuals suffering from metabolic syndrome and CAD, while performing regular exercise, showed impaired cerebral blood flow, which limits the benefit of exercise on cognitive health. Overtraining syndrome and associated brain impairment are also increasing the risk of high-intensity training. The relationship between metabolic syndrome, CAD, and brain abnormality is complex, which can be influenced by age, genetics, and lifestyle. Detection and management of these health problems through lifestyle modifications and medical interventions can control adverse effects on brain health in the context of athletic performance.

### 5.5. Congenital and Premature Heart Disease

Both congenital and premature heart disease have a long-term impact on neurological consequences. Even after surgical correction, congenital heart disease can disrupt normal neurodevelopment and leave lasting cognitive sequelae [177]. Similarly, premature or accelerated atherosclerosis manifests radiographically as white-matter hyperintensity and elevated diffusivity, correlating with faster cognitive aging [176,177]. Individual athletes with congenital or premature heart disease may develop reduced blood flow, neurological complications, sudden cardiac arrest, strokes, and seizures during intense physical activity, which require careful consideration and medical guidance.

**Figure 3:**
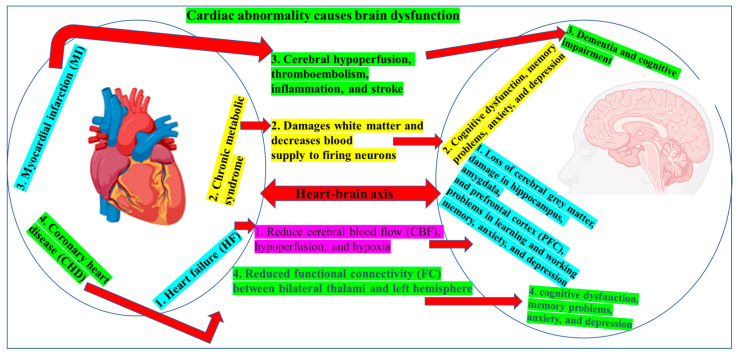
Cerebral consequences of cardiovascular pathology. Cardiac dysfunction, via reduced cardiac output, arrhythmias, or embolic events, diminishes cerebral blood flow, causing chronic hypoperfusion, tissue hypoxia, and white-matter injury. These cerebrovascular stressors foster cognitive decline, memory, and executive deficits, heightened dementia risk, and mood disturbances such as anxiety and depression.

Collectively, these findings highlight a cascade in which cardiac dysfunction initiates systemic inflammation, hormonal activation, and embolic or hypoxic injury that, in turn, damages neuronal circuits responsible for memory, executive control, and motor performance (Figure 3) [176,177]. For athletes, such neuro-cardiac reciprocity translates into diminished reaction time, impaired decision-making, and increased injury risk during play. Yet the molecular pathways linking cardiac disease to brain dysfunction in this population remain poorly defined, and prospective data on the immediate versus long-term cerebral effects of exercise-related cardiac abnormalities are scarce. Addressing these gaps will require integrative studies that pair advanced cardiac imaging, cerebral perfusion metrics, and neurocognitive testing with longitudinal follow-up to identify modifiable targets for intervention.

## 6. Conclusions

Neuro-cardiac crosstalk refers to the bidirectional, pathophysiological dialogue that links the central nervous and cardiovascular systems. It is no longer a theoretical construct but a clinically relevant reality that shapes outcomes after both sport-related head trauma and exercise-induced or inherited cardiac disease [146,147]. Data reviewed here show that TBI and SRC can precipitate acute autonomic imbalance, systemic inflammation, catecholamine toxicity, and inflammasome activation, culminating in arrhythmias, myocardial stunning, and longer-term atherogenic risk profiles in otherwise healthy athletes [19,20,22,23,24,98,111]. Conversely, common athletic cardiac disorders, i.e., HF, MI, atrial fibrillation, coronary artery disease, and metabolic syndrome, impair cerebral perfusion, promote micro-embolic injury, trigger neuro-inflammation, and accelerate cognitive decline and dementia [150,151,161,162,163]. These findings underscore three overarching messages.

1. Screening and surveillance must be bilateral. Pre-participation and post-injury evaluations should integrate basic neuro-cardiac metrics—heart rate variability, dynamic cerebral autoregulation, high-sensitivity troponin, and plasma inflammatory markers—to detect subclinical dysfunction in either organ early.
2. Rehabilitation should be network-based. Return-to-play protocols that combine graded aerobic exercise, autonomic retraining, and cognitive remediation hold promise for restoring both cardiac conditioning and cerebral plasticity after SRC or cardiac events.
3. Research priorities are clear. Prospective, sport-specific cohorts with unified neuro-cardiac phenotyping are needed to define dose–response curves for concussion burden and extreme training loads. Mechanistic trials should test whether targeting inflammasome signaling, catecholamine surges, or endothelial dysfunction mitigates long-term morbidity.

By framing brain and heart injuries as interconnected, rather than siloed, phenomena, sports neuroscience and cardiology can move toward precision prevention strategies that preserve the well-documented physical and psychosocial benefits of athletic participation while minimizing its emerging neuro-cardiac liabilities [59,60]. Closing the translational gap in this field will require interdisciplinary teams, shared data platforms, and longitudinal follow-up, but the reward—healthier brains and hearts across the athlete’s lifespan—justifies the effort. A comprehensive and timely management approach to sports-related injuries is recommended to optimize long-term athlete recovery. Although the scarcity of robust clinical evidence precludes definitive conclusions, the synthesized data offer a pertinent overview of sport-associated neurocardiac health and its implications for athletic performance. Ultimately, this work identifies a compelling avenue for future research, underscoring the need to elucidate the underlying mechanisms of neurocardiac pathophysiology.

## Figures and Tables

**Table 1:** Effects of Sport-Related Brain Injuries on Cardiovascular Function.

Type of TBI	Sport	Participants	Key Findings	Cardiovascular Relevance	Reference
Head injury	High schools and community sports	Healthy adolescents (11–18 years)	Glial fibrillary acidic protein decreases by 9.5% with each 1-year increase in age, adjusted for previous concussions	Biomarker changes may reflect neuroinflammation, which can influence autonomic function	[95]
Acute and sub-acute concussion	Athletics	Pediatric and collegiate	Contact sports exposure may increase brain age	Accelerated brain aging could predispose to autonomic dysfunction and CVD risk	[96]
Contact injury	Football	Pre-collegiate	Higher concussion risk for those starting football before age 12	Early exposure may lead to cumulative autonomic disruption	[97]
Sport and non-sport injury	Participant and non-participant	Children (5–12 years)	Similar injury recovery in both groups	Suggests concussion effects are consistent across contexts, including potential CVD risks	[99]
Contact and non-contact injury	Rugby	Retired players	Altered serum measurements (exosome size, t-tau, p-tau181, RBP-4) in retired athletes with concussion history	Biomarker changes may indicate chronic neuroinflammation, potentially affecting heart health	[101]
Repetitive head injury	High and low contact sports	High school athletes	Degraded neuropsychological results for high-contact athletes	Cognitive decline may correlate with autonomic dysfunction and altered HRV	[102]
Sport- and recreation-related brain injury	Different activities	Children (5–17 years)	Contact sports are more injury-prone	Higher concussion rates in contact sports may increase cumulative risk of autonomic imbalance	[103]
Sports- and recreation-related concussion	Different activities	Children (5–18 years)	Post-injury complexity and recovery depend on age	Age-related recovery differences may influence long-term autonomic and cardiovascular health	[104]

## Figures and Graphics

All graphics and figures were created using the Bio Render platform.
